# Sulfated non-anticoagulant heparin blocks Th2-induced asthma by modulating the IL-4/signal transducer and activator of transcription 6/Janus kinase 1 pathway

**DOI:** 10.1186/s12967-018-1621-5

**Published:** 2018-09-01

**Authors:** Mohamed A. Ghonim, Jeffrey Wang, Salome V. Ibba, Hanh H. Luu, Kusma Pyakurel, Ilyes Benslimane, Shaker Mousa, A. Hamid Boulares

**Affiliations:** 1 The Stanley S. Scott Cancer Center, LSU Health Sciences Center-New Orleans, 1700 Tulane Ave, New Orleans, LA 70112 USA; 2 The Department of Microbiology and Immunology, Faculty of Pharmacy, Al-Azhar University, Cairo, Egypt; 3 The Pharmaceutical Research Institute, Albany College of Pharmacy and Health Sciences, Rensselaer, NY USA; 4 Vascular Vision Pharmaceuticals Co., Rensselaer, NY USA

**Keywords:** Low-MW-heparins (LMWH), S-NACH, Th2 inflammation, Ovalbumin, Allergy, Therapeutic potential, Protein expression, Animal models of asthma

## Abstract

**Background:**

The efficacy of heparins and low-MW-heparins (LMWH) against human asthma has been known for decades. However, the clinical utility of these compounds has been hampered by their anticoagulant properties. Much effort has been put into harnessing the anti-inflammatory properties of LMWH but none have been used as therapy for asthma. Sulfated-non-anticoagulant heparin (S-NACH) is an ultra-LMWH with no systemic anticoagulant effects.

**Objective:**

The present study explored the potential of S-NACH in blocking allergic asthma and examined the potential mechanism by which it exerts its effects.

**Methods:**

Acute and chronic ovalbumin-based mouse models of asthma, splenocytes, and a lung epithelial cell line were used. Mice were challenged with aerosolized ovalbumin and administered S-NACH or saline 30 min after each ovalbumin challenge.

**Results:**

Sulfated-non-anticoagulant heparin administration in mice promoted a robust reduction in airway eosinophilia, mucus production, and airway hyperresponsiveness even after chronic repeated challenges with ovalbumin. Such effects were linked to suppression of Th2 cytokines IL-4/IL-5/IL-13/GM-CSF and ovalbumin-specific IgE without any effect on IFN-γ. S-NACH also reduced lung fibrosis in mice that were chronically-exposed to ovalbumin. These protective effects of S-NACH may be attributed to modulation of the IL-4/JAK1 signal transduction pathway through an inhibition of STAT6 phosphorylation and a subsequent inhibition of GATA-3 and inducible NO synthase expression. The effect of the drug on STAT6 phosphorylation coincided with a reduction in JAK1 phosphorylation upon IL-4 treatment. The protective effects of S-NACH treatment was associated with reduction of the basal expression of the two isoforms of arginase ARG1 and ARG2 in lung epithelial cells.

**Conclusions:**

Our study demonstrates that S-NACH constitutes an opportunity to benefit from the well-known anti-asthma properties of heparins/LMWH while bypassing the risk of bleeding. Our results show, for the first time, that such anti-asthma effects may be associated with reduction of the IL-4/JAK1/STAT6 pathway.

## Background

The pathogenesis of asthma is complex and multifactorial, affecting over 300 million people worldwide [1]. The disease is driven primarily by Th2 lymphocyte-mediated inflammation and is characterized by pulmonary eosinophilia, Th2 cytokines and allergen-specific IgE production, mucus hypersecretion, expression of inflammatory factors such as inducible nitric oxide synthase (iNOS), and airway hyperresponsiveness (AHR) [2, 3]. Although multiple therapeutic approaches are currently available, a considerable portion of the asthmatic population does not respond to standard therapies with the potential of developing uncontrollable exacerbations of the disease. Therefore, the need for novel approaches is immense and urgent.

Heparin and its low molecular weight derivatives (LMWH) are increasingly regarded to harbor anti-inflammatory traits that may be harnessed therapeutically against various inflammatory diseases including asthma [4–6]. In a clinical trial conducted almost two decades ago, an inhaled form of the LMWH enoxaparin was shown to prevent exercise-induced bronchoconstriction in asthmatics [7], which opened the possibility for these compounds to be used as therapeutic agents against some asthma traits. Recently, LMWH were reported to suppress allergen-specific IgE and Th2 cytokine production in mice chronically exposed to house dust mite extracts [8]. More recently, Patel et al. demonstrated that LMWH and montelukast can be combined into an inhalable particulate system that harbors efficient anti-inflammatory effects against ovalbumin (OVA)-induced asthma in rats [9]. However, different LMWH may have opposing effects as reported for enoxaparin and dalteparin [10]. Whereas enoxaparin reduced IL-4, IL-5, IL-13, and TNF-α in phytohaemagglutinin-treated peripheral blood mononuclear cells from asthmatics, dalteparin promoted an increase in the aforementioned cytokines. Although the underlying molecular mechanisms by which LMWH interfere with the process of inflammation has yet to be fully elucidated, some studies suggested that these heparins may bind directly to several inflammatory mediators leading to their neutralization [6, 11]. However, the major obstacle against the viability of LMWHs against asthma and other inflammatory disease is their anticoagulant property and the unacceptable risk for excessive bleeding [4–6].

Several studies have suggested that the anti-inflammatory aspect of LMWH may be different from its anticoagulant property [6]. This concept prompted a great deal of effort to formulate LMWH derivatives that maintain their anti-inflammatory property while losing the anticoagulant activity.

Indeed, Shastri et al. [12] isolated two fractions from the LMWH enoxaparin that were devoid of anticoagulant properties and demonstrated that these fractions inhibited T cell activation and reduced the release of IL-4, IL-5, IL-13 and TNF-α in peripheral blood mononuclear cells that were isolated from asthmatic individuals and were activated with phytohaemagglutinin, concanavalin-A or phorbol-esters. Sulfated non-anticoagulant LMWH (S-NACH) is an oxidized sulfated ultra-LMWH with limited to no systemic anticoagulant effects [13–15]. This property of S-NACH hypothetically would permit the administration of doses that surpass those that promote anticoagulation by the parent LMWH and thus would allow much greater anti-inflammatory outcomes without the risk of bleeding complications. In this study, we investigated whether S-NACH would efficiently block Th2-induced airway inflammation in acute and chronic animal models of asthma and determined whether this potential effect is associated with an interference with IL-4-induced signal transduction using an in vitro experimental model.

## Methods

### Animals

Six- to eight-week old C57BL/6J male mice were purchased from Jackson Laboratories (Bar Harbor, ME). Mice were maintained in a specific pathogen-free facility at LSU Health Sciences Center (LSUHSC) with unlimited access to sterilized chow diet and water.

### Ovalbumin (OVA) sensitization and challenge and enhanced pause (Penh) measurement

Mice were sensitized with 100 µg of Grade V chicken OVA (Sigma-Aldrich, St. Louis MO) mixed with 2 mg aluminum hydroxide in saline by intraperitoneal (*i.p.*) injection twice, once a week as previously described [16, 17]. Mice were then challenged with aerosolized 3% OVA for 30 min once for the acute model or 3 times per week for 4 weeks for the chronic model. Administration *i.p.* of S-NACH (water soluble) or vehicle (saline) was done 30 min after the OVA challenge. Mice were sacrificed 48 h later for bronchoalveolar lavage (BAL) or lung fixation and processing. Some mice were subjected to AHR measurements 24 h after the OVA challenge as described [16, 17].

### Organ recovery, tissue processing, cell staining, Th2 cytokine and OVA-specific IgE assessments, FACS analysis, and immunohistochemistry (IHC)

Lungs of sacrificed mice were fixed with formalin for histological analysis or subjected to BAL for cell counting and cytokine assessments as described [16, 17]. Tissue sections were subjected to hematoxylin and eosin (H&E), periodic acid-schiff (PAS), or trichrome staining using standard protocols as previously described. BAL fluids were subjected to cyto-spin, and the cells were stained with Diff-Quik (IMEB Inc, San Marcos, CA) for inflammatory cell assessments. Cytokine assessments were conducted using the Bio-Rad Bioplex system for mouse according to the manufacturer’s instructions. OVA-specific IgE levels were quantified with sandwich ELISA (Serotec, Raleigh, NC) as described previously [16, 17]. The hydroxyproline content in lung tissues was measured using a commercial assay kit (Quickzyme Biosciences, Netherlands) according to manufacturer’s instructions.

### Mouse splenocyte preparation, cell culture, treatments, and immunoblot analysis

The human lung cell line (A549) was purchased from ATCC (Manassas, VA). Splenocytes were isolated from C57BL/6 mice as described before [16, 17]. Treatment with IL-4, preparation of protein extracts, and immunoblot analysis were conducted using standard protocols. Antibodies used in this study were to the following proteins: GATA3 (Cell Signaling Technology, 5852, Danvers, MA) phospho(Y641)-STAT6 (Life Technologies, 700247, Carlsbad, CA), iNOS (Abcam, ab129372, Cambridge, United Kingdom), ARG1 (BD Biosciences, 610708, Franklin Lakes, NJ), ARG2 (Santa Cruz Biotechnology, sc-20151, Dallas, TX), phospho (Y1022, Y1023) JAK1 (ThermoFisher, 44-422G, Waltham, MA), JAK1 (Cell Signaling Technology, 3332), β-Actin (Santa Cruz Biotechnology, sc-47778), or GAPDH (Santa Cruz Biotechnology, sc-365062).

### Data analysis

All data are expressed as mean ± standard deviation (SD) of values from at least 6 mice per group, unless stated otherwise, or triplicate conditions when cells were used. All experiments were repeated at least two times. Prism software (GraphPad, San Diego, CA) was used to analyze the differences between experimental groups with one-way analysis of variance, followed by Tukey’s multiple comparison tests. For some results, analysis was conducted using unpaired Student’s *t*-test.

## Results

### Non-anticoagulant LMWH (S-NACH) efficiently reduces chronic asthma-like traits in a mouse model of the disease

**Fig. 1:**
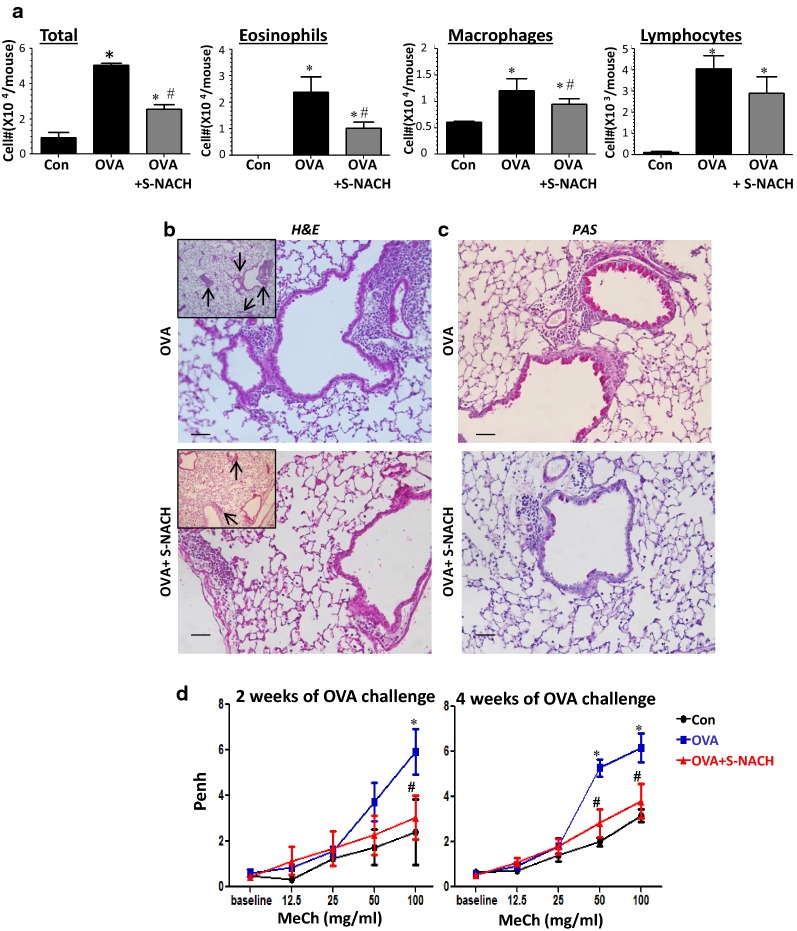
C57BL/6J mice were subjected to OVA sensitization followed by three challenges to OVA per week for 4 weeks or left unchallenged. A group of mice was administered, *i.p.*, 10 mg/kg of S-NACH or saline 30 min after OVA challenge. *Penh* was recorded 24 h after the last challenge of week 2 and week 4 using a whole body plethysmograph system before and after the indicated concentrations of aerosolized methacholine. Mice were sacrificed 48 h after the last challenge of week 4. Lungs were subjected to formalin fixation or BAL. **a** Cells of BALF were differentially stained, and total cells, eosinophils, macrophages, and lymphocytes were counted. Data are expressed as total number of cells per mouse. Data are mean ± SD of values from at least 6 mice per group. Lung sections from OVA-challenged mice that were treated with either saline or S-NACH were subjected to H&E (**b**) or PAS staining (**c**). For **b** and **c** Bar = 50 μm. Inserts represent a lower magnification of sections with arrows indicating sites of inflammation. **d** *Penh* measurements. Results are plotted as maximal fold increase of *Penh* relative to baseline and expressed as mean ± SEM, n = 6 mice per group. For **a** and **d** *difference from control unchallenged mice, *p* < 0.05; ^#^difference from OVA-challenged mice; *p* < 0.05

Multiple challenges of OVA-sensitized mice with aerosolized OVA promoted major infiltration of inflammatory cells into the lungs with a predominance of eosinophils and macrophages as well as lymphocytes, albeit to a lower extent. A single *i.p.* administration of 10 mg/kg S-NACH after each OVA challenge was sufficient to significantly reduce the overall recruitment of inflammatory cells to the lung. While eosinophils were the most sensitive cells to treatment, the effect of S-NACH on macrophage recruitment was moderate but statistically significant; however, its effect on T cell recruitment, although trended lower, was not statistically significant (Fig. 1a). Figure 1b displays representative H&E staining of lung sections from OVA-sensitized and challenged mice with or without S-NACH treatment. Hypersecretion of mucus is a major hallmark of asthma and a consistent trait that contributes to the severity and overall pathogenesis of the disease. PAS staining of lung sections from OVA-sensitized and challenged mice revealed a substantial mucus production accompanied by hyperplasia of epithelial cells (Fig. 1c). Such traits were dramatically reduced by S-NACH treatment. We then examined the effect of S-NACH treatment on lung function by measuring the enhance pause (*Penh*) using whole body plethysmography. While this method does not measure airway resistance, it does provide an indication of the sensitivity of the lungs to bronchoconstriction mediated by methacholine inhalation. The assessment was conducted after 2 and 4 weeks into the OVA challenge protocol. Figure 1d shows that S-NACH treatment protected against the bronchoconstrictive effect of methacholine at the 100 mg/kg dose. Interestingly, when *Penh* was measured after 4 weeks of the OVA challenge, S-NACH treatment provided an even better protection, and the response of the treated mice was similar to that observed in control, unchallenged mice.

### S-NACH protects against chronic asthma-like traits by reducing Th2 cytokines and OVA-specific IgE production without affecting IFN-γ levels

**Fig. 2:**
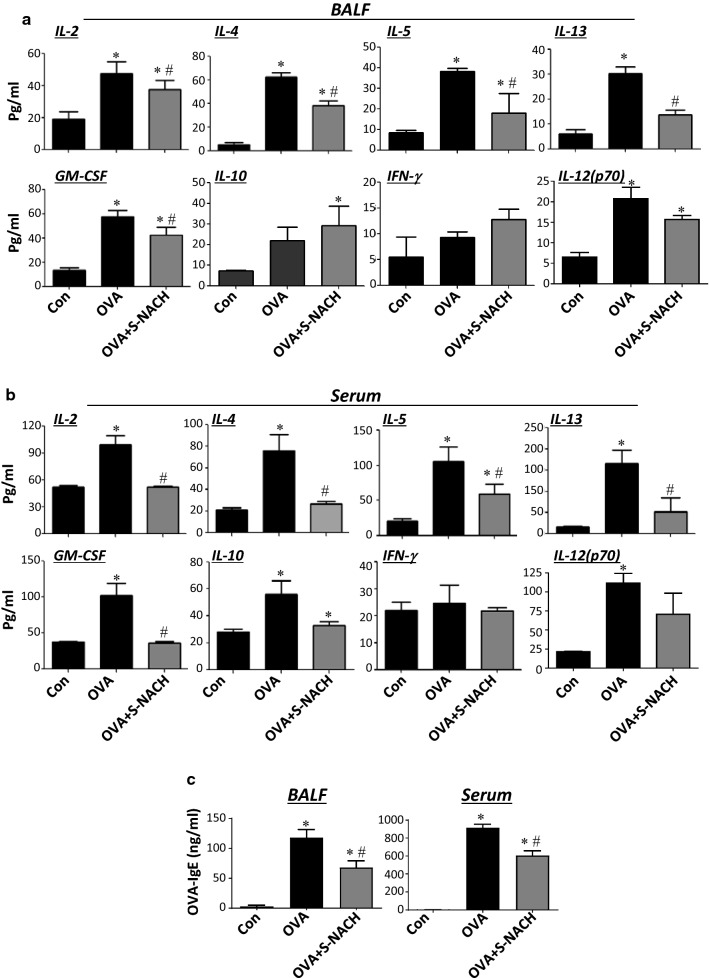
BALF (**a**) and sera (**b**) collected from the different mouse groups described in Fig. 1 were assessed for different Th1 and Th2 cytokines using a multiplex assay. **c** BALF and sera were assessed for OVA-specific IgE with ELISA. Data are mean ± SD of values from at least 6 mice per group. *Difference from control unchallenged mice, *p* < 0.05; ^#^difference from OVA-challenged mice; *p* < 0.05

Several Th2 and Th1 cytokines were assessed in sera and BALF. Figure 2a shows that chronic exposure to OVA induced, as expected, substantial levels of IL-2, IL-4, IL-5, IL-13, and GM-CSF assessed in BAL fluids of treated animals. Treatment with S-NACH significantly reduced the production of these cytokines in the lungs of OVA-challenged mice. Interestingly, while S-NACH slightly enhanced the levels of the anti-inflammatory cytokine IL-10, it did not exert any effect on IL-12 or IFN-γ. Figure 2b shows that S-NACH treatment was effective at reducing systemic inflammation because the effect of the drug on the measured pro-inflammatory cytokines was very potent. In fact, the levels of IL-2, IL-4, IL-5, IL-13, and GM-CSF were comparable to those detected in control naïve animals. While S-NACH treatment reduced the anti-inflammatory cytokine IL-10, it did not affect the levels of the other anti-inflammatory cytokine IL-15 (data not shown). Moreover, S-NACH treatment did not affect the levels of IL-12 or the basal levels of IFN-γ detected in both sera and BAL fluids of OVA-challenged mice.

Because allergen-specific IgE production is a critical component of allergic responses including asthma, we examined the effect of S-NACH treatment on lung and systemic OVA-specific IgE using ELISA. S-NACH treatment reduced OVA-specific IgE levels by ~ 50% in BAL fluids of OVA-challenged mice and to a lesser extent systemically (Fig. 2c). These results suggest that the protective effect of S-NACH against asthma-like traits may be associated with a modulation of Th2 cytokines and allergen-specific IgE production.

### S-NACH administration provides an excellent protection against acute challenge to OVA in mice

**Fig. 3:**
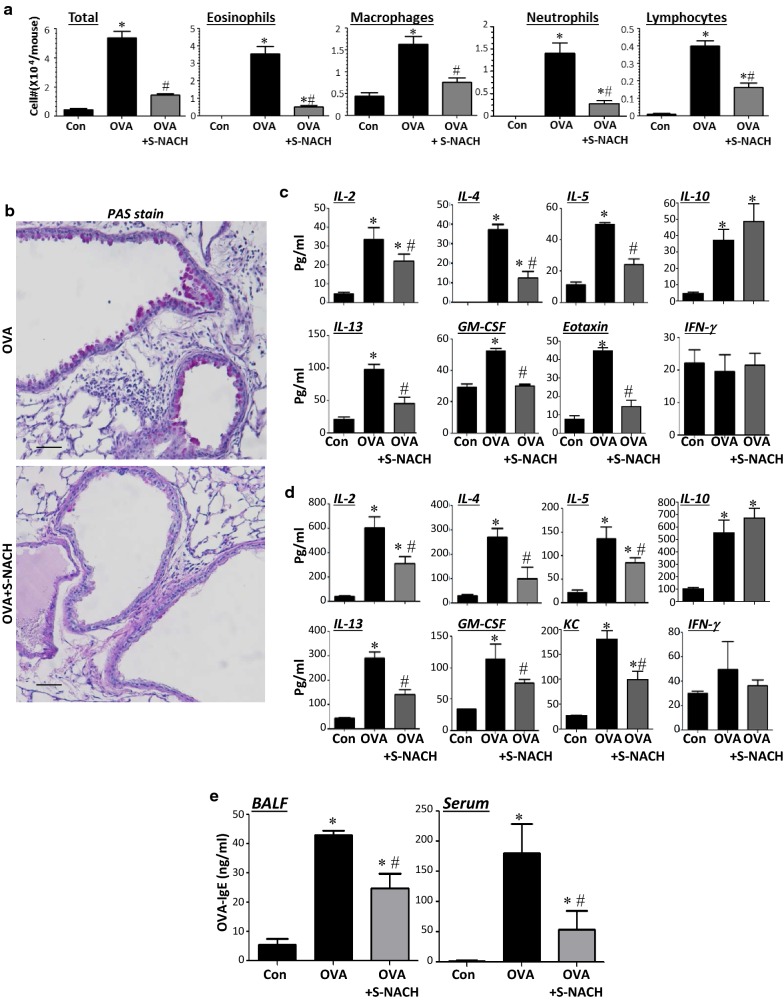
Mice were subjected to OVA sensitization followed by a single challenge or left unchallenged. Mice were administered, *i.p.*, 10 mg/kg S-NACH or saline 30 min after OVA challenge. Mice were then sacrificed 48 h later and lungs were subjected to formalin fixation or BAL. **a** Cells of BALF were differentially stained, and total cells, eosinophils, macrophages, and lymphocytes were counted. Data are expressed as total number of cells per mouse. Data are mean ± SD of values from at least 6 mice per group. **b** Lung sections from OVA-challenged mice that were treated with either saline or S-NACH were subjected to PAS staining; Bar = 50 μm. BALF (**c**) and sera (**d**) collected from the different mouse groups were assessed for different Th1 and Th2 cytokines using a multiplex assay. **e** BALF and sera were assessed for OVA-specific IgE with ELISA. Data are mean ± SD of values from at least 6 mice per group. For (**a**), (**c**), (**d**), and **e** *difference from control unchallenged mice, *p* < 0.05; ^#^difference from OVA-challenged mice; *p* < 0.05

Acute manifestation of asthma symptoms is an important aspect of the disease because it often requires a different line of treatment [1]. We therefore examined the efficacy of S-NACH against an acute exposure to an allergen using a model of the condition. Figure 3a shows that a single *i.p.* administration of 10 mg/kg S-NACH was highly effective at reducing lung cellularity with a prominent effect on eosinophils, macrophages, and neutrophils. Unlike in the chronic model, S-NACH significantly reduced the number of lymphocytes. These effects were accompanied by a pronounced reduction in mucus production and the associated hyperplasia of epithelial cells as shown in Fig. 3b. An examination of Th2 and Th1 cytokines in BAL fluids (Fig. 3c) and sera (Fig. 3d) of OVA-challenged animals revealed that the increase in IL-2, IL-4, IL-5, IL-13, and GM-CSF upon an acute exposure to OVA was significantly reduced upon treatment with S-NACH. Consistent with the effect of S-NACH treatment in the chronic model, it did not exert any inhibitory effects on IL-10 or IFN-γ. The levels of the chemokine KC (CXCL1) increased upon OVA challenge in sera of animals, S-NACH treatment significantly reduced the levels of this chemokine. S-NACH treatment also reduced OVA-specific IgE levels in both BAL fluids and sera of OVA-challenged mice (Fig. 3e). Overall, our findings suggest that the protective effect of S-NACH against asthma-like traits may be associated with a modulation of Th2 cytokines and OVA-specific IgE both locally in the lung and systemically upon an acute or chronic exposure to OVA.

### S-NACH reduces lung fibrosis as measured by collagen deposition in lungs of chronically exposed mice

**Fig. 4:**
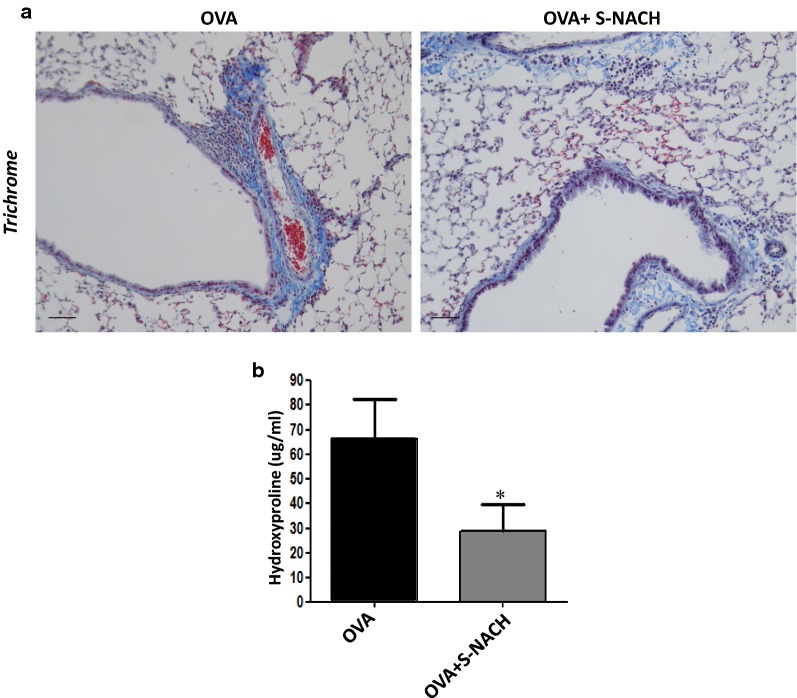
Lung sections of mice that were subjected to chronic OVA challenge with or without S-NACH administration were subjected to trichrome staining (**a**) Bar = 50 μm. Equal weight of lung tissue from the two groups was processed for hydroxyproline assessment as described in the “Methods” section. **b** The hydroxyproline contents were measured using a commercial assay kit. Data are mean ± SD of values from at least 6 mice per group. *Difference from OVA-challenged mice, *p* < 0.05

Collagen deposition is a hallmark of lung remodeling upon chronic exposure to allergens. Given the observation that S-NACH blocked airway inflammation and mucus production upon chronic exposure to OVA, we examined whether the drug also blocked pulmonary fibrosis. Figure 4a shows that chronic OVA challenge induced substantial collagen deposition in lungs of mice. Such collagen deposition was significantly lower in S-NACH treated mice that were subjected to repeated exposures to OVA. This finding was confirmed by measuring the level of hydroxyproline (Fig. 4b), an established measure for collagen content in tissues.

### The protective effect of S-NACH against Th2-mediated inflammation and fibrosis may be associated with a modulation in the IL-4/STAT6 pathway

**Fig. 5:**
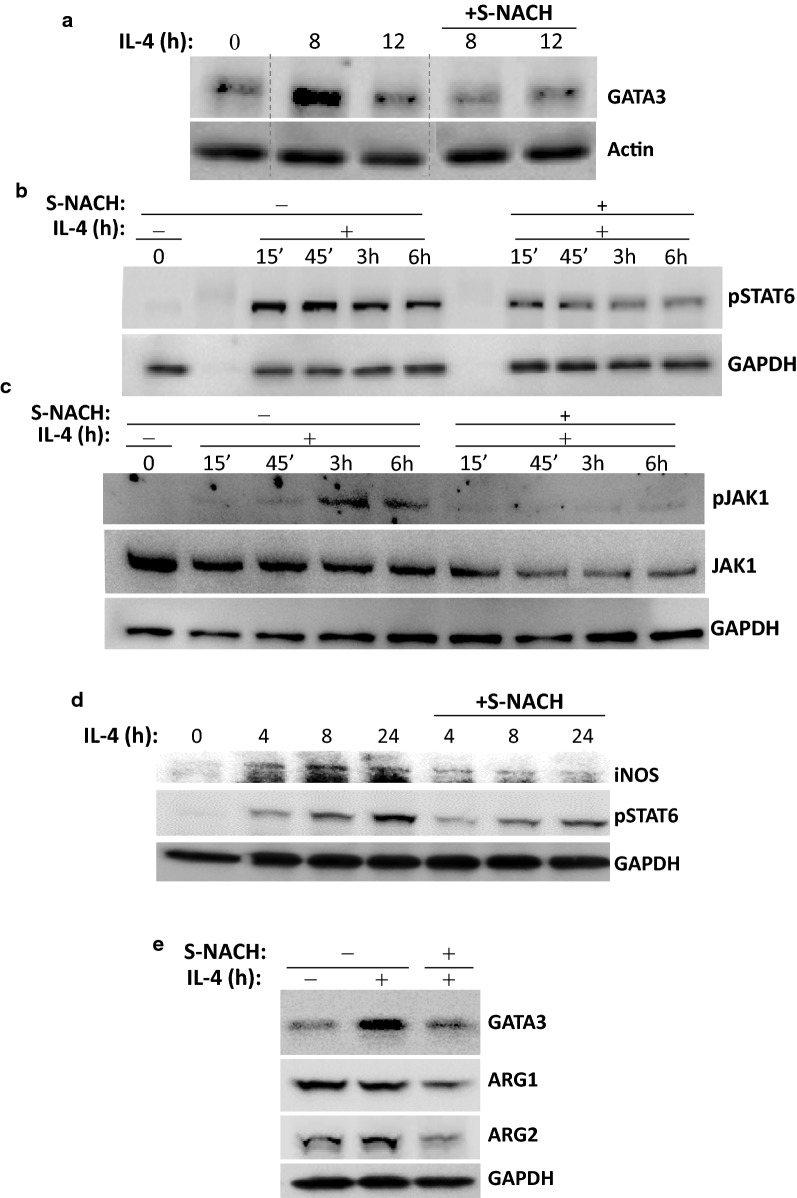
**a** Splenocytes isolated from naïve C57BL/6 mice were stimulated with 10 ng/ml of mouse IL-4 for 0, 8, or 12 h in the absence or presence of 50 μg/ml S-NACH. Protein extracts were then prepared and subjected to immunoblot analysis with antibodies to GATA-3 or actin. **b** Splenocytes were treated as in (**a**) but collected after 0 min, 15 min, 45 min, 3 h, or 6 h of treatment. Protein extracts were then prepared and subjected to immunoblot analysis with antibodies to the phosphorylated form of STAT6 (p-STAT6) or GAPDH. **c** Protein extracts from splenocytes that were treated in a manner similar to those described in (**b**) were subjected to immunoblot analysis with antibodies to the phosphorylated form of JAK1 (p-JAK1), JAK1, or GAPDH. **d** A549 cells were treated with 10 ng/ml of human IL-4 for the indicated time intervals in the presence or absence of 50 μg/ml S-NACH. Protein extracts were prepared and subjected to immunoblot analysis with antibodies to iNOS, pSTAT6, or GAPDH. **e** A549 cells were treated as in (**d**) for 24 h. Protein extracts were subjected to immunoblot analysis with antibodies to GATA-3, ARG1, ARG2, or GAPDH

The IL-4/JAK/STAT6 pathway is a major driver of many of the processes associated with Th2 inflammation [2, 3]. STAT6 is phosphorylated upon IL-4-receptor stimulation by JAK kinases and then translocated to the nucleus after homodimerization. STAT6 serves as a transcription factor for various genes including *gata*-*3* [18, 19]. It is noteworthy that GATA-3 is the master regulator of the *IL*-*4/IL*-*5/IL*-*13* cytokine locus [20]. We thus examined whether the mechanism by which S-NACH interferes with Th2 cytokine production stems from a modulation of IL-4/JAK/STAT6 signal transduction and GATA-3 expression. Figure 5a shows that an in vitro treatment of mouse splenocytes with IL-4 induced an increase in GATA-3 above baseline levels as assessed with immunoblot analysis, and that treatment with 50 μg/ml S-NACH prevented such increase. Figure 5b shows that the modulation of GATA-3 by S-NACH treatment may be associated with reduced phosphorylation of STAT6 at tyrosine-641. The reduction in STAT6 phosphorylation in S-NACH-treated cells coincided with a decrease in the activity of JAK1 as assessed by its phosphorylation level at tyrosine-1022 and -1023 (Fig. 5c).

We have recently shown a close association between iNOS expression and lung inflammation and fibrosis in a model of severe asthma [21, 22]. Furthermore, an increase in arginase activity was shown to contribute to airway remodeling in animals [23]. Figure 5d shows that IL-4 induced modest levels of iNOS in the human lung epithelial cell line A549 and that treatment of the cells with S-NACH reduced the expression of the protein. This effect of the drug coincided with reduced phosphorylation of STAT6. Figure 5e shows that S-NACH treatment was also effective at reducing IL-4-induced GATA-3 expression in A549 cells as well as the endogenous levels of ARG1 and ARG2.

## Discussion

Our results show that the sulfated LMWH, S-NACH, was highly effective at blocking different asthma traits in mice. The anti-inflammatory properties of S-NACH were observed in both an acute and a chronic model of the disease. We also provided evidence that S-NACH may be blocking Th2 inflammation, in part, by blocking IL-4-mediated signal transduction. Indeed, S-NACH reduced JAK1 phosphorylation with a consequent reduction in STAT6 phosphorylation, which resulted in significant reduction in the expression of GATA-3, the master regulator of the *IL*-*4/IL*-*5/IL*-*13* gene locus. We also found that S-NACH blocks fibrosis potentially through a downregulation of iNOS, ARG1, and ARG2. These results may represent an important advance in considering the potential use of S-NACH and the concept of non-anticoagulant LMWH in anti-inflammatory diseases such as asthma. It is noteworthy that unlike the conventional LMWH, S-NACH can be envisioned to be used at relatively higher doses than its parent compounds. It is important to note that in the chronic model, S-NACH was delivered *i.p.* only 3 time a week; we predict that if the drug were administered on a daily basis and perhaps in an inhaled form, the protective effects would be much more pronounced.

We acknowledge that our study is not sufficient to provide a comprehensive understanding of the mechanism by which S-NACH mediates the modulation of Th2-inflammatory responses. We do know that LMHW have been tested in asthma models similar to the two used in the current study [9, 24, 25]; however, it is not clear whether S-NACH exerts its effects exactly as do the parent compounds. It is possible that the modifications may play a role in the modulation process. However, Shastri et al. showed that two fractions from the LMWH enoxaparin without any targeted modification reduce Th2 cytokine release in peripheral blood mononuclear cells from asthmatic individuals [12] suggesting that the moiety within LMWH that exhibits the anticoagulant property is different from the one that displays the anti-inflammatory trait. Undoubtedly, determining the exact mechanism of action of S-NACH requires additional experimentation. This study is the first to report that S-NACH and maybe the parent LMWH interfere with IL-4-associated signal transduction by reducing JAK1 and STAT6 phosphorylation. S-NACH was very efficient at reducing GATA-3 in IL-4-treated splenocytes and the lung epithelial cell line A549, this modulation may be directly linked to the effect of S-NACH on STAT6 phosphorylation given the fact that activated STAT6 drives the expression of GATA-3. The effect on GATA-3 may in turn explain the simultaneous effects of S-NACH treatment on IL-4, IL-5, and IL-13 in mice acutely or chronically challenged with OVA. These results are consistent with those reported by Huang et al. [26] using a house dust mite extract-based model of asthma. Unlike that study, which reported a modulatory effect of LMWH on both Th1 and Th2 cytokines, our study showed that S-NACH was more selective against Th2 cytokines because it did not affect IFNγ levels after OVA exposure either acutely or chronically. To study the mechanism by which S-NACH reduces Th2 cytokine production in immune cells, a splenocytes-based cell culture model was utilized. We show that S-NACH treatment was effective at preventing the increase in GATA3 above baseline levels. It is important to note that we cannot deduce from these results that S-NACH prevents Th2 cell differentiation. While stimulation with IL-4 alone increased GATA3 above baseline levels in this model, these levels may not be sufficient to promote Th2 cell differentiation as TCR stimulation is required [27]. Additional studies are required to determine whether S-NACH treatment directly interferes with Th2 cell differentiation by incubating naïve CD4^+^ T cells in Th2-skewing culture conditions including TCR-stimulating antibodies (anti-CD3 and anti-CD28) and antibodies to IL-12 in the presence of IL-4 [17]. Another possible mechanism worth mentioning that was not addressed in the current study is the potential of S-NACH interfering with antigen presentation or B cell function given the findings that the drug was efficient at reducing IgE production both in the acute and chronic models of asthma.

S-NACH treatment appeared to block fibrosis in the chronic asthma model, which we believe may be associated with its anti-inflammatory effect. However, we recently showed that for inflammation and fibrosis to co-exist, expression of iNOS is required [21]; indeed, in a model similar to the one used in the current study, iNOS gene deletion blocked lung fibrosis but not inflammation. The persistence of inflammation in iNOS^−/−^ mice that were chronically exposed to OVA was attributed, in part, to increased levels of ARG1 and ARG2. S-NACH treatment blocked expression of IL-4-induced expression of iNOS in lung epithelial cells and reduced the basal levels of Arg1 and Arg2. It is tempting to correlate the effects of sulfated LMWH to the effects on iNOS and arginases; however, more experimentation is necessary to decipher the exact mechanism by which S-NACH exerts its effects.

One of the limitations of this study is the utilization of *Penh*. Although *Penh* measurement does not exactly reflect lung resistance, it provides an indication that S-NACH treatment may protect lung function during allergen exposure. AHR can be affected by numerous factors including inflammation and lung smooth muscle cell contractility [28]. Given our results, we can only conclude that S-NACH protected against lung dysfunction, in part, by reducing inflammation. To examine whether S-NACH affects lung smooth muscle cell function, more precise experimentation is required. An additional limitation of the present study is the use of OVA as an inducer of Th2 inflammation. Although OVA is widely used for proof of concept experiments, future studies need to include the use of allergens that are more relevant to human asthma, such as house dust mite extracts cockroach extracts, and spores from *Aspergillus* species [29].

## Conclusion

The efficacy of LMWH in blocking some or all asthma traits is certain given the positive results of numerous studies exploring this possibility in clinical trials and preclinical models of the disease The initial studies showing such potential were reported several decades ago [30, 31]; see detailed review by Mousavi et al. [4]. However, the utility of these heparins as a monotherapy or adjuvant therapy has been severely hampered by the anticoagulant aspect of the drugs and the high risk of uncontrolled bleeding. The results attained in this study demonstrate the efficacy of S-NACH against asthma and present S-NACH as a viable alternative to take advantage of the known effects of LMWH against asthma in humans.

## Abbreviations

LMWH: low molecular weight heparin
S-NACH: sulfated-non-anticoagulant heparin
iNOS: inducible NO synthase
STAT6: signal transducer and activator of transcription 6
Arg1: arginase 1
OVA: ovalbumin
AHR: airway hyperresponsiveness
*Penh*: enhance pause
